# Emerging Mutations Potentially Related to SARS-CoV-2 Immune Escape: The Case of a Long-Term Patient

**DOI:** 10.3390/life11111259

**Published:** 2021-11-18

**Authors:** Loredana Capozzi, Domenico Simone, Angelica Bianco, Laura Del Sambro, Valeria Rondinone, Lorenzo Pace, Viviana Manzulli, Michela Iacobellis, Antonio Parisi

**Affiliations:** 1 Istituto Zooprofilattico Sperimentale della Puglia e della Basilicata, Via Manfredonia 20, 71121 Foggia, Italy; loredana.capozzi@izspb.it (L.C.); domenico.simone@izspb.it (D.S.); angelica.bianco@izspb.it (A.B.); laura.delsambro@izspb.it (L.D.S.); valeria.rondinone@izspb.it (V.R.); lorenzo.pace@izspb.it (L.P.); viviana.manzulli@izspb.it (V.M.); 2 Cytopathology Department of the Hospital Di Venere, Via Ospedale di Venere 1, 70131 Bari, Italy; michela.iacobellis@asl.bari.it

**Keywords:** COVID-19, SARS-CoV-2, genomic surveillance, whole genome sequencing, Pangolin lineage, genome mutations, seroneutralization assay, long-term patient

## Abstract

SARS-CoV-2 isolates from long-term COVID-19 patients play a significant role in understanding the mechanisms of infection and virus persistence. This study describes a SARS-CoV-2 isolate from a 53-year-old woman from Apulia (Italy), who was COVID-19 positive for approximately four months. In this paper we aimed to investigate any potential correlation between genetic mutations and clinical features of this case of infection. The viral isolate was assigned to lineage B.1.177.51 through whole-genome sequencing (WGS) and harbored a novel set of mutations on the Spike protein (V143D, del144/145 and E484K); furthermore, seroneutralization assays showed impaired response of the surveyed strain to BNT162b2 (Comirnaty) Pfizer/BioNTech vaccine-induced (average reduction of 70%) and convalescent sera (average reduction of 19.04%), when compared to VOC P.1. This study highlights the importance of genomic surveillance for the management of the COVID-19 pandemic, the relevance of monitoring of emerging SARS-CoV-2 mutations in all lineages, and the necessity of testing the response of emerging variants to available therapies and vaccines.

## 1. Introduction

The Severe Acute Respiratory Syndrome Coronavirus 2 (SARS-CoV-2) was identified for the first time in December 2019 as the cause of an outbreak of a severe respiratory infection, namely, Coronavirus Disease 2019 (COVID-19). As of early November 2021, there have been 249,743,428 confirmed cases of COVID-19, including 5,047,652 deaths worldwide [1].

While methods for detection of the virus are well established and rely on quantitative reverse transcription polymerase chain reaction (RT-PCR), understanding of molecular mechanisms related to SARS-CoV-2 infection is still quickly evolving, with the aim of translating the huge amount of knowledge produced in effective prevention, therapy, and containment strategies [2,3,4,5]. In this perspective, genomic surveillance through whole-genome sequencing (WGS) provides a fast and reliable tool to track patterns of viral spreading and emergence of new mutations which may confer a selective advantage to the virus, as observed with enhancement of transmissibility of VOC 202012/01 ([6], accessed on 17 May 2021). Lineage B.1.177 (dubbed “the Spanish variant”), first detected in Spain in early summer 2020, has become the predominant variant in many EU countries and rapidly originated new sub-lineages, including B.1.177.51. To date, there is no evidence supporting an increased transmissibility of these variants.

In the past few months, emerging mutations in the spike (S) glycoprotein have been constantly under surveillance due to the prominent role of this protein in the viral infection, with special concern for S:E484K since it occurs in the receptor binding domain (RBD) [7]. Furthermore, this mutation has arisen in multiple lineages independently and has been identified in a case of reinfection [8]. Therefore, it has been speculated that S:E484K could provide an evolutionary advantage for SARS-CoV-2 lineages to escape the host immune response. Similarly, it has been shown that independent deletion events followed by transmission, such as the loss of S glycoprotein residues 144/145, could improve viral resistance to antibody neutralization [9].

Investigation of SARS-CoV-2 strains from “long-haulers” COVID-19 patients offers the opportunity to gain deep understanding of mechanisms of infection and persistence of the virus. Here we describe the case of a peculiar viral genotype isolated from a long-term COVID-19 patient. This isolate, assigned to Pangolin lineage B.1.177.51 and harboring a unique set of mutations on the gene encoding the S protein, will be referred to as B.1.177.51:TS (B.1.177.51 from this study) throughout this paper. The effects of these mutations on the efficiency of vaccine-induced and convalescent sera were assessed through a seroneutralization comparative assay including different viral genotypes (B.1, P.1).

## 2. Case Description

On 13 November 2020, a 53-year-old woman tested positive to SARS-CoV-2 through nasopharyngeal swab, with mild symptoms (fever). Afterwards, the patient tested positive two more times, then, on November 19, she was admitted to Policlinico Hospital (Bari, Apulia) with high fever and cough. At the hospital she was stabilized and then sent home where, after three days, she worsened with low oxygen saturation, fever, and cough. Therefore, she was hospitalized again and treated for a month with antibiotic therapy. During the subsequent hospitalization, the patient tested positive three more times, then discharged on 6 December with positive swab and fever. She was treated at home with antibiotics, cortisone, and chloroquine. However, eight additional tests performed between December 2020 and mid-February 2021 did not show clearance of the virus in the patient. She tested negative for the first time on 8 March 2021. Subsequently the patient underwent chest X-ray and showed scarring of pneumonia on the left.

## 3. Materials and Methods

### 3.1. Specimen Collection and Testing

Genotyping analysis was performed on a nasopharyngeal swab collected from the patient on 16 February 2021 and tested for SARS-CoV-2 infection at Di Venere Hospital laboratory (Carbonara di Bari, Italy). The sample was stored in COPAN UTM^®^ Universal Transport Medium (COPAN Diagnostics, Inc., Murrieta, CA, USA) and sent to the Molecular Biology Laboratory of the Istituto Zooprofilattico Sperimentale della Puglia e della Basilicata (Putignano, Apulia region, Italy) for sequencing.

Viral RNA was purified using the QIAamp Viral RNA Minikit (Qiagen, Hilden, Germany). The presence of SARS-CoV-2 was confirmed by multiplex real-time reverse transcription polymerase chain reaction (rRT-PCR) test using Gene Finder™ COVID-19 Plus Real Amp Kit (OSANG Healthcare Co., Ltd., Anyang-si Gyeonggi-do, South Korea). Real-time PCR was performed on Applied Biosystems 7500 Real-Time PCR System (software version 2.3).

### 3.2. cDNA Synthesis and Viral Genome Amplification

cDNA synthesis was performed using Luna Script RT Super Mix Kit (New England Biolabs, Ipswich, MA, USA), according to manufacturer’s instructions. The synthesized cDNA was used as template for direct amplification performed in two multiplexed PCR reactions, to generate ~400 bp amplicons tiled across the genome. PCR amplifications were carried out using the multiplex primers sets, described by the ARTIC Network (V3 nCov-2019 primers: https://artic.network/ncov2019, accessed on 7 June 2021), and Q5 Hot Start High-Fidelity 2X Master Mix (New England Biolabs, Ipswich, MA, USA) as previously described [10].

### 3.3. Library Preparation and Whole Genome Sequencing

Sequencing library preparation approach was adapted from the ARTIC (Advancing Real-Time Infection Control) V3 Network protocol and Illumina Nextera DNA Flex Library protocol (Illumina, San Diego, CA, USA). PCR amplicons obtained were combined and purified with Ampure XP beads (Beckman Coulter, Brea, CA, USA). Purified sample was used for library preparation with Illumina DNA Prep sample preparation kit (Illumina), as previously described [11]. Sequencing was performed on Illumina MiSeq platform (Illumina) using the MiSeq Reagent Kit v2, 2 × 250 paired-end cycles. A total of 134,397 paired-end reads were obtained from the sequencing run.

### 3.4. Sequence Data Analysis

A consensus sequence of the SARS-CoV-2 specimen was reconstructed from raw data with the SARS-CoV-2 Illumina GeNome Assembly Line pipeline (v1.1.0, https://github.com/jaleezyy/COVID-19-signal, accessed on 16 November 2021). Briefly, raw reads were trimmed with trimgalore (https://www.bioinformatics.babraham.ac.uk/projects/trim_galore, accessed on 16 November 2021) and mapped to the SARS-CoV-2 reference genome (AC: NC_045512.2) with bwa-mem [12]. Primers were removed from aligned reads with ivar trim, then the consensus genome was obtained with ivar consensus [13].

### 3.5. Phylogenetic Analysis

Lineage was assigned with Pangolin (https://github.com/cov-lineages/pangolin/blob/master/github.com/cov-lineages/pangolin, accessed on 16 November 2021), by the lineage definition updated to 14 May 2020. Phylogenetic analysis aimed at confirming the viral genome lineage included all 949 genomes from our region (Apulia) available from GISAID on 27 May 2021. A multiple sequence alignment of the dataset was performed with the MAFFT FFT-NS-2 algorithm with default parameter settings [14]. GTR+I+G4 was chosen with MODELTEST [15] as the most suitable model of nucleotide substitution for phylogeny reconstruction. A maximum likelihood tree was then computed with IQ-TREE2 [16] using the GTR+I+G4 model and ultrafast bootstrapping with 1000 replicates, using the Wuhan-Hu-1 sequence (GenBank accession number NC_045512.2) as outgroup. The resulting phylogenetic tree was displayed and decorated with iTOL [17] and is reported in Supplementary Figure S1.

### 3.6. Seroneutralization Assays

The seroneutralization assays on the virus isolate were performed at the biosafety level 3 (BSL-3) laboratory of the Istituto Zooprofilattico Sperimentale of Puglia and Basilicata (Foggia, Italy) on Vero E6 cells, as previously described [18]. The neutralizing antibody titer was defined as the highest serum dilution at which no cytopathic effect (CPE) breakthrough in any of the testing wells was observed.

We evaluated SARS-CoV-2 resistance to neutralization using serum from 18 participants obtained 7–10 days after receipt of the second dose of mRNA COVID-19 vaccine-BNT162b2 (Comirnaty) Pfizer/BioNTech and convalescent serum obtained from 16 patients after infection with COVID-19. These patients had not received any vaccine dose by the time of serum sampling.

Blood samples were collected from 18 randomly selected vaccinated healthcare workers. Serum neutralizing antibody titers were compared for B.1.177.51:TS and for two other viral strains. The first was a wild-type strain of SARS-CoV-2 belonging to the B.1 lineage (GISAID accession number: EPI_ISL_568579), isolated on March 2020 in Italy and provided by Prof. Fausto Baldanti, Fondazione IRCCS Policlinico San Matteo (Pavia, Italy). The second one was an isolate belonging to the Brazilian P.1 lineage.

The seroneutralization test was carried out simultaneously with 16 serum samples from immunized patients who were considered suitable for plasma donation. The titer of neutralizing antibodies from hyperimmune sera was compared against B.1.177.51:TS and the B.1 lineage strain.

## 4. Results

**Figure 1:**
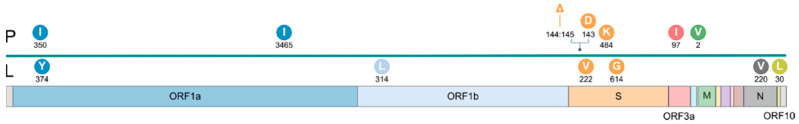
Amino acid mutations identified in the B.1.177.51:TS genome. Only ORFs harboring mutations are labeled. L = lineage-defining mutations (defined as mutations occurring in >75% genomes assigned to that lineage); P = private mutations. The figure was adapted from graphical reports generated through the outbreak.info website.

The mean genome coverage depth of the sample was 1575X. The reconstructed genome has been deposited to the GISAID repository (https://www.gisaid.org/, accessed on 17 November 2021) with the accession ID EPI_ISL_1112236. Nextclade and Pangolin analyses assigned the surveyed genome sequence to clade 20E (EU1) and lineage B.1.177.51, respectively. The reconstructed genome harbored all the amino acid changes characteristic of the assigned lineage (Figure 1).

Seven additional changes, unrelated to B.1.177.51 lineage, were also detected, with three of them occurring in the S protein (V143D, del144/145, E484K; Figure 1). Further confirmation of lineage assignment was inferred from a phylogenetic tree including all SARS-CoV-2 sequences from Apulia available as per 27 May 2021 (Supplementary Figure S1). The complete list of amino acid mutations of B.1.177.51:TS is available in Supplementary Table S1. 

**Figure 2:**
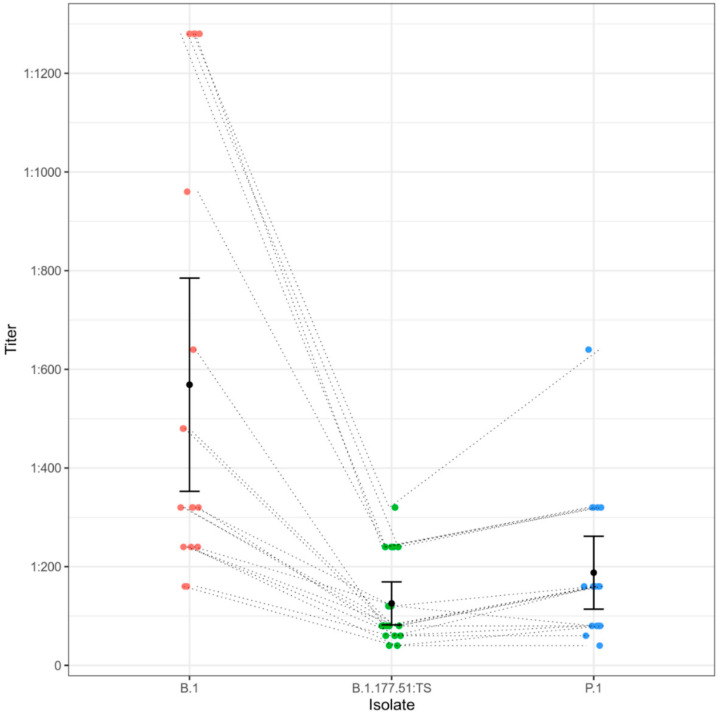
Evaluation of serum response from 18 healthcare workers vaccinated with BNT162b2 (Comirnaty) Pfizer/BioNTech vaccine against four different SARS-CoV-2 strains. Mean titers with 95% confidence intervals are shown for serum response against each strain. Dashed lines show how each individual serum response changes in different isolates.

Efficiency of serum samples from 18 vaccinated health workers was tested against B.1.177.51:TS and compared with samples representative of two other lineages (B.1 and P.1). Mean titer value for B.1.177.51:TS was 1:125 (95% CI, 40–320); for B.1, 1:568 (95% CI, 160–1280); and for P.1, 1:187 (95% CI, 40–640). Neutralizing antibody efficiency was reduced, on average, by 78% against B.1.177.51:TS and by 70% against the P.1 strain, compared to the antibody titers reported for B.1 isolate (Figure 2).

All neutralizing antibody titer values are shown in Supplementary Table S2.

**Figure 3:**
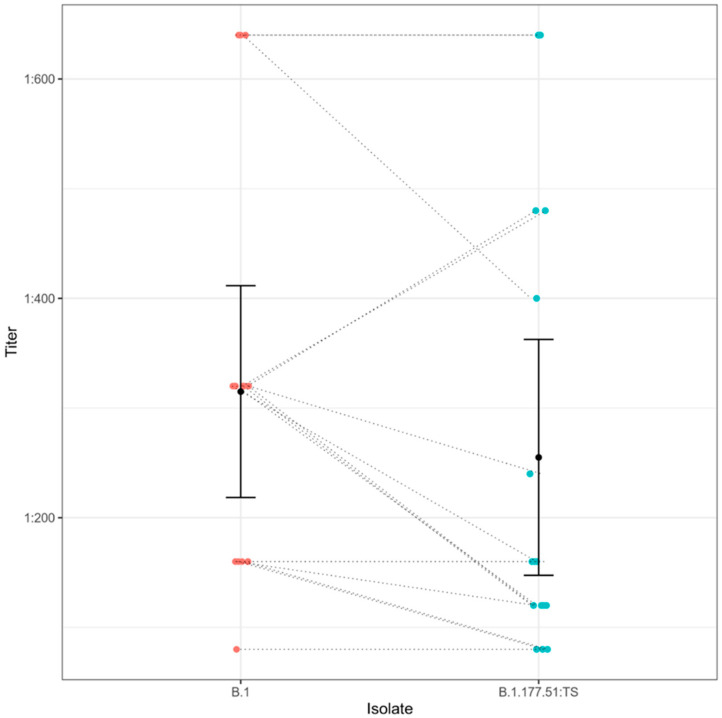
Evaluation of serum response from 16 convalescent individuals against two different SARS-CoV-2 strains. Mean titers with 95% confidence intervals are shown for serum response against each strain. Dashed lines show how each individual serum response changes in different isolates.

Efficiency of serum samples from 16 convalescent individuals was tested against B.1.177.51:TS and compared with a sample from lineage B.1. Mean titer value for B.1.177.51:TS was 1:255 (95% CI, 80–640); for B.1, 1:315 (95% CI, 80–640). This assay reported an antibody efficiency average reduction of 19.04% against B.1.177.51:TS when compared to the B.1 isolate (Figure 3).

The seroneutralization values observed with the hyperimmune sera against B.1.177.51:TS and the B.1 isolate are shown in Supplementary Table S3.

## 5. Discussion

In this paper we report the case of a 53-year-old woman who had tested positive for SARS-CoV-2 infection for almost four months, from November 2020 to March 2021. Genome analysis assigned the isolate to the B.1.177.51 lineage. B.1.177, belonging to clade 20E (EU1), was first detected in Spain in early summer 2020 [19] and spread throughout European countries and globally [20], including Italy where it became a major circulating lineage [5].

However, the surveyed SARS-CoV-2 genome, namely B.1.177.51:TS, also harbored three mutations in the S-protein which are not related to lineage B.1.177.51, namely S:V143D, S:del144/145, and S:E484K. While S:del144 is very frequent (>90%; source: https://outbreak.info, accessed on 17 November 2021) in several B.1 sub-lineages, including the variant of concern (VOC) B.1.1.7, this is not the case for the double deletion S:del144/145 (<0.5%). Deletions in this region of the S protein reportedly suppress the binding of the S-protein to a specific class of monoclonal antibodies [21], although it still allows binding of antibodies targeting the Receptor Binding Domain (RBD). S:E484K has been shown to confer an increased potential to escape the host immune response [7]. S:V143D is a rare mutation (<0.5% of genomes in GISAID) and in B.1.177.51:TS occurs as a consequence of the deletion of the six nucleotides leading to del144/145. However, its location in the S-protein and the shift of chemical properties (hydrophobic to polar) may suggest a role in changing the affinity of the Spike protein to target sites and/or antibodies.

B.1.177.51:TS showed on average a reduction of response to both vaccine-induced (78%) and convalescent (19.04%) sera. Based on the peculiar genetic arrangement of B.1.177.51:TS and the detected immune response, we cannot rule out that the long-term positivity of the patient to the virus may be related to this specific genotype. Indeed, these mutations might have emerged in response to the selective pressure exerted during the long-term infection. To support this hypothesis, we can consider that the viral specimen was sampled from the patient three months after the diagnosis and, as per 18 May 2021, no other genome from Apulia or Italy with the B.1.177.51:TS genotype is available in the GISAID repository. This hypothesis is surely limited by the lack of a greater amount of viral specimens from the same patient at different time points and by a deeply representative sequencing effort in the same geographic area. However, previous studies have shown that both S:E484K and deletion in positions S:144 and S:145 have arisen independently multiple times [8,21] with possible links to long-term infections. Seroneutralization assays suggested that B.1.177.51:TS impaired the neutralizing efficiency of both vaccine-induced and convalescent sera. In particular, the vaccine-induced sera performed even worse on B.1.177.51:TS when compared to VOC P.1 (Figure 2), although both types of serum may retain sufficient response against all surveyed strains.

In previous studies, convalescent sera were shown to have a neutralizing efficiency against SARS-CoV-2 strains harboring the S:N501Y [22] and S:E484K mutations, S:69/70 deletion [23] VUI lineage B.1.617 mutations [24], although a slightly lower response was seen against VUI B.1.617 and S:E484K. The variation in neutralizing efficiency between convalescent plasma and vaccinated individuals sera was also investigated: both neutralized a S:N501Y mutant strain [25], while the B.1.351 variant escaped convalescent and vaccine-induced sera [26].

This study is limited to the analysis of a single viral strain related to long-term cases of infection, tested against sera on small cohorts of vaccinated healthcare workers and convalescent patients. Furthermore, we were limited to sequencing the isolate from only one of several nasopharyngeal swabs sampled from the patient. This did not allow us to understand whether the particular set of genetic mutations was acquired by the virus over the four months during infection, or whether the woman had been infected with this mutated isolate from the beginning. Despite the limitations, the data obtained from this study provide important insights into particular cases of infection, such as this one characterized by significant viral persistence in the host. Our findings agree with previous reports and point out that convalescent hyperimmune sera may foster a greater neutralizing effect than the antibody response after the two doses of Pfizer-BioNTech vaccine BNT162b2. From a public health perspective, whole-genome sequencing (WGS) of SARS-CoV-2 allows real-time tracking of virus global spread patterns, providing important data for genomic epidemiology investigations. Furthermore, this study highlights the importance of genomic surveillance for the management of the COVID-19 pandemic and the relevance of molecular monitoring of emerging SARS-CoV-2 mutations in all lineages, especially those occurring on the gene encoding the S-protein. Therefore, we suggest that the appearance of novel mutations should be monitored in all lineages (even those not considered VOCs) through exhaustive and carefully planned genomic surveillance programmes. Our findings also suggest the need of coupling genomic surveillance and assessment of traits of novel variants (e.g., transmissibility, virulence, immune escape) as a valuable support for the pandemic management. In this perspective, monitoring of circulating SARS-CoV-2 variants should be coordinated as a global effort in order to maximize advances and translation of knowledge in the field.

## 6. Conclusions

We described a SARS-CoV-2 isolate which, despite being assigned to lineage B.1.177.51, harbored a set of mutations potentially increasing its persistence within the patient. The results of the tests performed in this study provided some relevant insights into the potential correlation between the genetic characteristics of SARS-CoV-2 isolates and the clinical conditions of affected patients. In addition, the detection of mutations in strains assigned to non-VOC variants, may reveal important viral characteristics. This paper highlights the importance of genomic surveillance as a primary tool for pandemic management and development of impactful prevention, containment, and therapy strategies.

## Data Availability

Data available in a publicly accessible repository that does not release DOIs: The datasets obtained in this study have been made publicly available. These data can be found on the official GISAID repository, here: [https://www.gisaid.org/] (accessed on 17 November 2021). The Accession ID of the sequences obtained in this study is EPI_ISL_1112236.

## Supplementary Materials

The following are available online at https://www.mdpi.com/article/10.3390/life11111259/s1, Figure S1: Phylogenetic tree of all Apulian SARS-CoV-2 genomes available through GISAID as per 18 May 2021. The tree was computed with IQ-TREE 2 as detailed in the Methods section. Genome B.1.177.51:TS is marked with a star, Table S1: The complete list of the amino acid changes in the B.1.177.51:TS genome, Table S2: The table shows the neutralizing antibody titer values for the 18 sera (HW1 to HW18) obtained from vaccinated healthcare workers, tested against strains B.1, B.1.177, B.1.177.51 with E484K mutation, P.1, respectively, Table S3: The table shows the neutralizing antibody titer values for the 16 hyperimmune sera (P1 to P16) obtained from patients, tested against strains B.1, B.1.177.51 with E484K mutation, respectively.
