# Supplementary material for: Emerging Mutations Potentially Related to SARS-CoV-2 Immune Escape: The Case of a Long-Term Patient
Source: Life (Basel). 2021 Nov 18;11(11):1259. doi: 10.3390/life11111259 (PMC8625042; doi:10.3390/life11111259)
Supplement: Supplementary file 1 [file life-11-01259-s001.zip › life-1443683-supplementary.pdf]

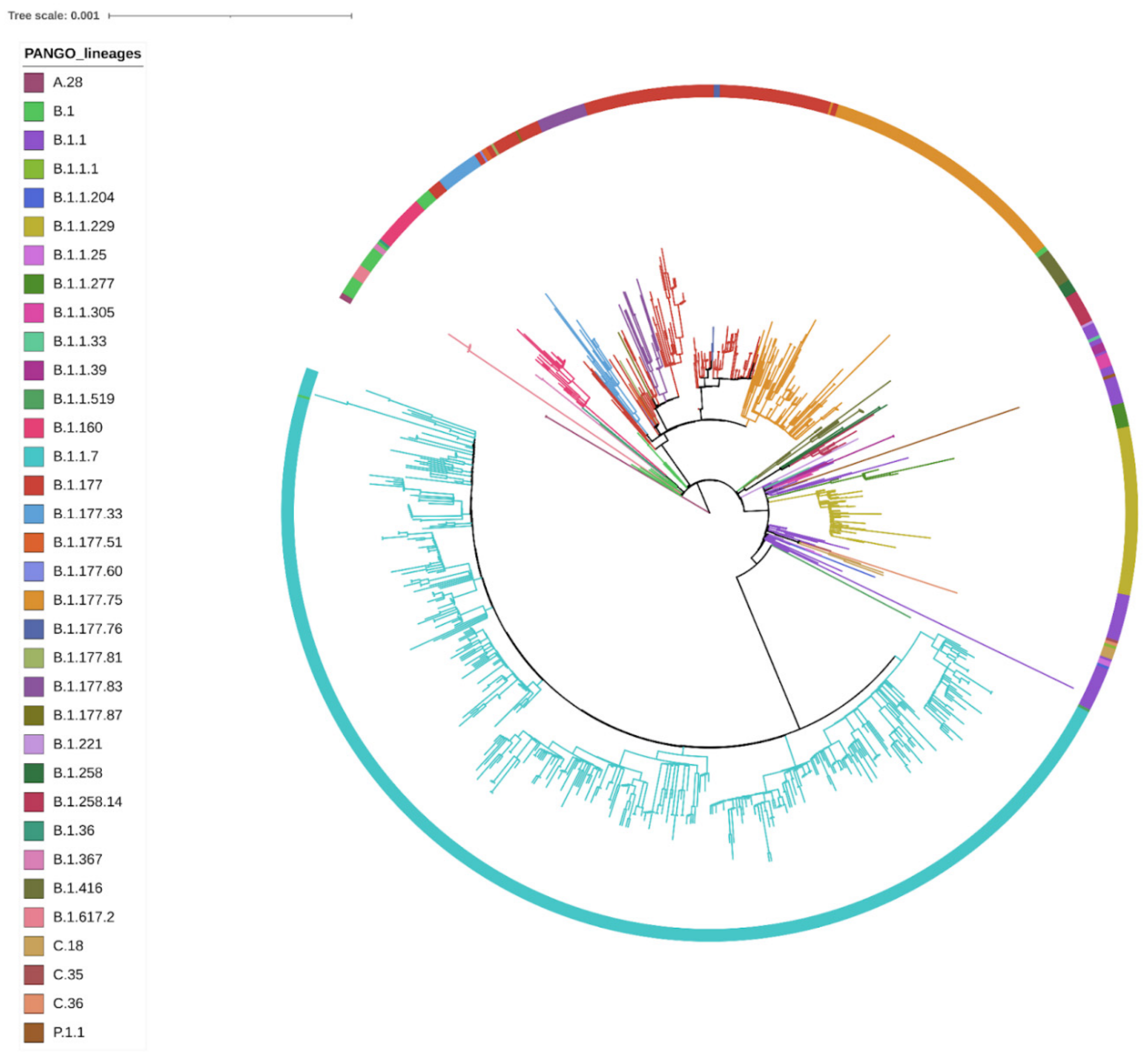

Supplementary Figure S1. Phylogenetic tree of all Apulian SARS-CoV-2 genomes available through GISAID as per May 18th, 2021. The tree was computed with IQ-TREE 2 as detailed in the Methods section. Genome B.1.177.51:TS is marked with a star.

Genome position	Nucleotide change	Gene	Aminoacid change	Lineage defining
241	C241T		-	
445	T445C	ORF1a	-	
1314	C1314T	ORF1a	T350I	private mutation
1385	C1385T	ORF1a	H374Y	B.1.177.51
3037	C3037T	ORF1a	-	
6286	C6286T	ORF1a	-	

10658	G10658A	ORF1a	V3465I	private mutation
14408	C14408T	ORF1b	P314L	B.1.177.51
21255	G21255C	ORF1b	-	
21989	G21989-TTTATT	S	V143D	private mutation
21989	G21989-TTTATT	S	del144/145	private mutation
22227	C22227T	S	A222V	B.1.177.51
23212	G23212A	S	E484K	private mutation
24303	A24303G	S	D614G	B.1.177.51
25681	G25681A	ORF3a	V97I	private mutation
26527	C26527T	N	A220V	B.1.177.51
26801	C26801G	M	-	
27944	C27944T	ORF8	-	
28932	C28932T	M	A2V	private mutation
29645	G29645T	ORF10	V30L	B.1.177.51

Supplementary Table S1. The complete list of the aminoacid changes in the B.1.177.51:TS genome.

ID	B.1	B.1.177.51:TS	P.1
HW1	1:320	1:80	1:160
HW2	1:480	1:80	1:80
HW3	1:320	1:80	1:160
HW4	1:640	1:80	1:160
HW5	1: 1280	1:240	1:320
HW6	1:320	1:120	1:160
HW7	1:1280	1:240	1:320
HW8	1:240	1:120	1:80
HW9	1:160	1:60	1:80

HW10	1:960	1:240	1:320
HW11	1:320	1:60	1:160
HW12	1: 1280	1:240	1:320
HW13	1:1280	1:320	1:640
HW14	1:240	1:40	1:80
HW15	1:240	1:80	1:160
HW16	1:160	1:40	1:40
HW17	1:480	1:80	1:80
HW18	1:240	1:60	1:60

Supplementary Table S2. The table shows the neutralising antibody titre values for the 18 sera (HW1 to HW18) obtained from vaccinated healthcare workers, tested against strains B.1, B.1.177, B.1.177.51 with E484K mutation, P.1, respectively.

ID	B.1	B.1.177.51:TS
P1	1:160	1:120
P2	1:160	1:80
P3	1:160	1:80
P4	1:320	1:120
P5	1:320	1:120
P6	1:320	1:120
P7	1:320	1:160
P8	1:320	1:480
P9	1:80	1:80
P10	1:320	1:480
P11	1:160	1:160
P12	1:320	1:240
P13	1:640	1:400
P14	1:640	1:640
P15	1:160	1:160
P16	1:640	1:640

Supplementary Table S3. The table shows the neutralising antibody titre values for the 16 hyperimmune sera (P1 to P16) obtained from patients, tested against strains B.1, B.1.177.51 with E484K mutation, respectively.
